# Case Report: Upper Thoracic Purely Extradural Spinal Meningioma With Nerve Root Attachment: A Case Report and Literature Review

**DOI:** 10.3389/fsurg.2022.918094

**Published:** 2022-06-16

**Authors:** Zhao-Lin Wang, Jian-Hui Mou, Dong Sun, Peng Liu

**Affiliations:** Department of Orthopaedics, China-Japan Union Hospital of Jilin University, Changchun, China

**Keywords:** 2766 extradural spinal meningioma, nerve root, hemilaminectomy, rare, case report

## Abstract

**Introduction:**

We describe a case of purely extradural spinal meningioma (EDSMs) with nerve root attachment and present the clinical profiles, radiological findings, operative management, and follow-up data, along with a literature review. This case study is purely extradural spinal meningioma attached to the nerve root, and the available literature review discusses the diagnosis and treatment. Purely epidural spinal meningiomas are extremely rare, and only a few clinical reports are published on this disease. Although epidural meningioma is a benign tumor, the recurrence rate is higher when compared with ordinary meningioma.

**Case study:**

A 39-year-old male complained of chest and back pain with ring-shaped radiations to the precordial area and numbness in both the lower extremities. After a long walk, he felt disharmony in both his lower limbs. Magnetic resonance imaging showed a mass located on the left posterolateral side of the T1–T3 spinal cord and exhibited a dumbbell-type growth outward at the level of the T2–T3 intervertebral foramen. On the left side of the thoracic, a hemilaminectomy procedure was performed. The tumor was found in the ventral side of the left nerve root of T1 and was surrounded by the left nerve root of T2. To obliterate the tumor, the T2 nerve root was severed. The patient was advised to come for the follow-up on the 3rd, 6th, and 12th months postoperatively, and there were no complaints or signs of recurrence.

**Conclusion:**

Purely extradural spinal meningioma with nerve root attachment is rare and has no clinical symptoms and image findings. To completely resect the lesion and avoid recurrence, the affected nerve root, epidural fat tissue, and nerve root sheath should be extensively resected and burned, coagulating the adjacent ventral posterior longitudinal ligament.

## Introduction

Spinal meningiomas are commonly considered primary spinal tumors, which account for up to 25%–46% of all primary spinal tumors and 7.5%–12.7% (1, 2) of all meningiomas. Most of the meningiomas are located in the thoracic region confined to the intradural extramedullary (3), but meningiomas that occur extradural are extremely rare (<10%). Although purely epidural meningiomas are extremely rare, few clinical reports evidence these conditions (4, 5). Meningiomas around the nerve roots are difficult to distinguish from schwannomas because both are intradural extramedullary tumors and are associated with neurofibromatosis; hence, they are difficult to distinguish. Moreover, schwannomas mainly originate from the Schwann cells in the dorsal root ganglion; this clues that intraganglionic glial cells are potential cells that serve as the origin for schwannomas and estimated that approximately 10% to 15% form dumbbell-shaped tumors spreading along the nerve root (6). Due to their location and mode of slow growth, it is easy to misdiagnose. The symptoms are usually mild and often reflect other similar morbidities, thus affecting the formulation of therapeutic management (5, 7–9). We report a case of purely extradural spinal meningioma (T1–T3) located in the upper thoracic region and an appropriate review of the literature.

## Case Report

**Figure 1:**
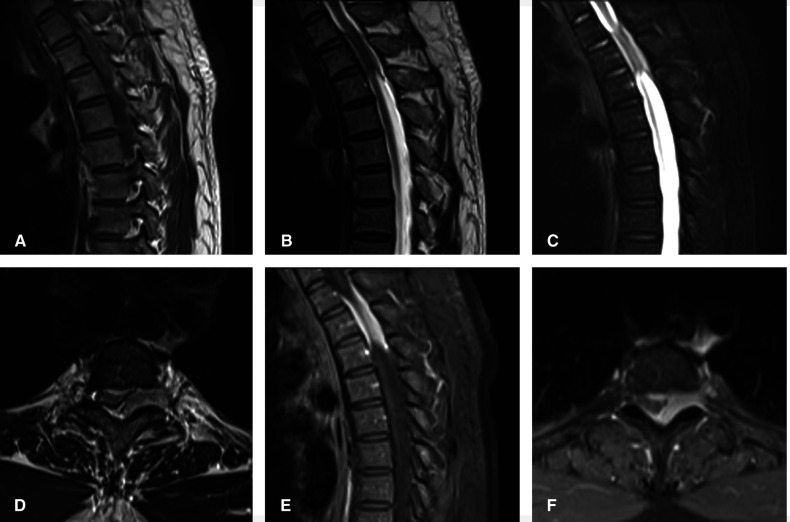
Preoperatively, MRI of thoracic vertebrae showed strip-like equal T1 signals in the spinal canal at the level of T1–T3 vertebrae, low and equal T2 signals, and high signals in fat-suppression sequence. The contrast-enhanced T1 weighted phase showed obvious uniform enhancement. The meningioma located on the left posterolateral side of the spinal cord, and growth outward at the level of T2–3 intervertebral foramen. (**A**) Sagittal T1-weighted, (**B**) Sagittal T2-weighted, (**C**) Sagittal T2-weighted fat-suppression sequence, (**D**) axial T2-weighted, Sagittal (**E**) and axial (**F**) of the contrast-enhanced T1 weighted phase.

**Figure 2:**
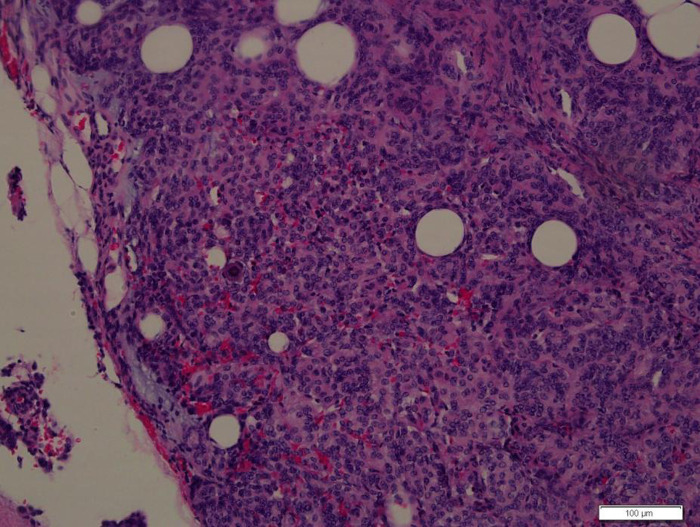
Photomicrographs of the surgical specimens showing sheets of meningothelial cells with oval nuclei characteristic of meningioma (H&E, ×200).

A 39-year-old healthy male complained of pain in his left chest and back, the pain persisted for 11 days, with ring-shaped radiations to the precordial area, and numbness in both the lower extremities continued for 9 days. The skin sensation below the costal margin was weakened, and there was only a sense of banding. After a long walk, the patient could feel a sense of disharmony in both lower limbs. The most prevalent signs were patellar clonus, ankle clonus, and Babinski sign, which were significantly positive. MRI of thoracic vertebrae showed a strip-like structure equal to T1 signals in the spinal canal at the level of T1–T3 vertebrae, low and equal T2 signals, and high signals in the fat-suppression sequence. The contrast-enhanced T1 weighted phase showed a uniform enhancement, with clear borders and wide base attachments close to the dural membrane. The meningioma was located on the left posterolateral side of the spinal cord, and dumbbell-type growth was outward at the level of the T2–T3 intervertebral foramen (Figure 1). Computerized tomography (CT) with a value of 67HU showed a soft tissue mass in the spinal canal at the level of the T1–T3 vertebral body. A spot-like calcification density was observed, and the spinal cord was compressed and moved to the right. The enhanced scan showed moderately uneven enhancement. The CT-mediated biopsy of the T2–T3 intervertebral foramina lesion showed meningioma (Figure 2). Immunohistochemistry showed that Vim, E-cad, S-100, D2-40, PR, and EMA were (+), whereas, Ki67 (5%) and CK were (−). The left thoracic hemilaminectomy was performed under general anesthesia. The operation was extended to the maximum possibility to increase the exposure field and ensure the minimum displacement of the spinal cord during the operation. At the same time, the left intervertebral joint bone of T2/T3 was resected, and the resected bone was preserved; later, the bone graft was prepared by calcining and trimming. The tumor was gray-colored, appeared fish-like structure, rich in blood vessels, located on the left posterolateral side of the dura mater, and grew to the anterolateral side of the spinal cord but did not exceed the midline of the ventral side. The tumor penetrated deep in and grew outwards, located in the ventral side of the left nerve root of T1 and surrounded by the left nerve root of T2. With a nerve stripper exploration, the tumor could completely strip from the T1 root and dura mater and get separated from the T2 nerve root, which was unclear. To remove the tumor completely, the T2 nerve root was severed, the sleeve was burned, and a sufficient hemostatic was applied that was covered with a gelatin sponge to autologous with the bone grafted. Postoperative pathology showed meningioma, immunohistochemical EMA, PR, S-100 (+), Ki-67 (1%), and E-Cadherin (+). The patient’s sense of banding disappeared immediately after the surgery, and there was no uncoordinated walking even after attempting a long walk. Cerebrospinal fluid leakage occurred on the 3rd day postoperatively, but there were no symptoms of hypotensive cranial pressure such as headache, nausea, and vomiting. He was encouraged to walk daily, suggested to lie on his right side, and change his dressing daily. The cerebrospinal fluid leakage recovered on the 10th day of the postoperative procedure, and the patient was discharged on the 13th day of the postoperative procedure. The patient was advised to come for the follow-up on the 3rd, 6th, and 12th months postoperatively, and there were no further complaints observed. MRI showed no recurrence of the mass, no compression of the spinal cord, and no instability of the thoracic spine.

## Discussion

Purely extradural spinal meningioma is unusual, and tumor with nerve root attachment is extremely rare. A systematic literature review was undertaken by searching for articles on PubMed, EBSCO, and ScienceDirect, using the keywords “Purely extradural spinal meningioma.” Reviewing the literature, a few studies have described purely extradural meningioma (Table 1).

**Table 1:** Summary of purely extradural spinal meningiomas reported.

Author	Cases	Age/Gender	Location	Presentations	Bony changes	Root Involved	Surgical treatment	Histology	Adjuvant treatment	Follow up
H. Milz, et al., (10)	1	70Y/F	T5	Motor deficits, Sensory disturbance	Normal	Not reported	Gross total resection, Dural resection	Meningothelial	No	Not reported
	1	45Y/F	T6	Motor deficits, Sensory disturbance	Normal	Not reported	Gross total resection, Dural resection and grafting	Psammomatous	No	Not reported
Dimitris Zevgaridis, et al., (5)	1	75Y/F	T11–T12	Revealed occasionally	Normal	No	Gross total resection	Meningioma	No	No recurrence
Messori A, et al., (11)	1	14Y/F	C5–C7	Motor deficits, Sensory disturbance, Sphincer dysfuction	Normal	No	Gross total resection, Dural resection and grafting	Meningothelial meningioma with abundant psammoma bodies	No	Neurologically improved, No recurrent
Jayshree Tulit, et al., (12)	1	42Y/F	T5	Pain, Motor deficits, Exaggerated ankle reflexes	Normal	Yes	Gross total resection, Rhizotomy (left T5), Fusion and fixation (T4-5)	Meningioma	No	Neurologically improved
Liang Wu, et al., (13)	12	5M, 7F	Cervical spine (9), Cervicothoracic spine (1), Thoracic spine (2)	Motor deficits (10), Sensory disturbance (12), Sphincter dysfunction (2), Hoarseness (1)	Normal	6	Gross-total resection (4), Subtotal resection (8), Laminectomy (7), Laminoplasty (5), Dura was coagulated or removed and reconstructed	Psammomatous (7), Meningothelial (4), Transitional (1)	Low-dose radiation therapy (2)	Neurologically improved, No recurrence in 11 cases, 1 case recurrenced and underwent a second operation for removing the residual lesion completely
Savardekar A, et al., (8)	1	35Y/F	C3–C6	Pain, Motor deficits, Sensory disturbance	Invaded the C4 and C5 lateral spinous processes	No	Subtotal resection	Meningothelial	No	Asymptomatic
	1	23Y/F	T5	Pain, Motor deficits, Sensory disturbance, Bowel and bladder involvement	Normal	No	Gross total resection	Meningioma	No	Neurologically improved, No recurrent
Ben Nsir A, et al., (9)	1	70Y/M	T5	Pain, Motor deficits, Sensory disturbance	T5 vertebral body was in T2 hypersignal	No	Gross total resection with posterolateral approach, Dural was cauterized	Meningioma	50 Gy radiation therapy	Recurrent with progression including T5 vertebral body and posterior mediastinum, Gross total resection with posterolateral transthoracic approach, T5 vertebral body removal and stabilization, Neurologically improved, No recurrent
Bettaswamy G, et al., (4)	1	50Y/M	C1–C4	Pain, Motor deficits, Sensory disturbance, Bladder disturbance	Normal	No	Subtotal resection, Dural was cauterized	Meningothelial	No	Neurologically improved, No recurrent
	1	41Y/M	C3–C7	Pain, Motor deficits, Sensory disturbance	Normal	No	Gross total resection	Meningothelial	No	Neurologically improved, No recurrent
Dehcordi SR, et al., (14)	1	39Y/F	T3–T4, T5–T6	Motor deficits, Sensory disturbance	Bone erosion	No	Gross total resection, Dural was cauterized	Meningothelial	No	Neurologically improved, No instability, No recurrent
Sharad Pandey, et al., (15)	1	18Y/M	T7–T9	Pain, Motor deficits, Sensory disturbance, Constipation and urinary incontinence	Normal	No	Gross total resection	Psammomatous	No	Neurologically improved
Demir MK, et al., (16)	1	26Y/F	T9–T11	Pain, Motor deficits, Sensory disturbance	Normal	Not reported	Gross total resection	Meningothelial meningioma with psammoma bodies	No	Not reported
Ishita Pant, et al., (2)	1	50Y/M	C5–C7	Motor deficits, Sensory disturbance	Marrow signal abnormality in C6–C7 vertebral bodies, Left C5-6 and C6-7 neuroforaminal widening	No	Gross total resection	Meningothelial	No	Neurologically improved
Anna Lois Lai, et al., (3)	1	35Y/M	C1–C4	Pain, Motor deficits, Sensory disturbance	C1-4 neuroforaminal widening and scalloping	No	Subtotal resection, Anterior decompression and fusion (C2–C3)	Meningothelial	No	Neurologically improved, No recurrent
Hammad Ghanchi, et al., (17)	1	40Y/M	T6, L1	Pain, Motor deficits	Sclerosis and bony effacement of vertebral body without destruction	No	Gross total resection	Meningioma	No	Neurologically improved, No recurrent
Isabel Tulloch, et al., (18)	1	45Y/F	T5–T7	Pain, Motor deficits, Sensory disturbance	Normal	Yes	Gross total resection, Dural was cauterized	Chordoid meningioma	No	Neurologically improved, No recurrent
Cher Shui, et al., (19)	1	66Y/F	T3–T5	Motor deficits, Sensory disturbance	Calcification without bony destruction	Yes	Subtotal resection, Rhizotomy (left T5), Fusion and fixation (T3-T5)	Meningothelial (occasional psammomatous calcification)	No	Neurologically improved
Benjamin Pommier, et al., (20)	1	74Y/F	C6-C7	Pain, Motor deficits	Normal	No	Gross total resection	Psammomatous	No	Neurologically improved

*F, Female; M, male; Y, years*.

### Prevalence

Meningioma is a primary central nervous system (CNS) tumor originating from the meninges, a membrane surrounding the brain or spinal cord. Although it is often benign, there is still a high incidence of its occurrence. In the recent WHO guidelines in 2016, 3 grades of meningiomas were approved; they justified that this grading system would correlate with the risk of recurrence and overall survival and could better guide the treatment strategy. Hence, it is essential to know the incidence of each grade where WHO declared that 80.5% of all meningiomas have benign histology and indolent behavior. Further, it was estimated that grades II and III make up 17.7% and 1.7% of meningiomas, respectively. It is considered that Ki-67 proliferation index of >4% and >20% have an increased risk of recurrence and mortality, respectively (21–23)

There is no difference observed related to gender specificity, and it is considered that the occurrence is four times as common in women as in men, with a peak incidence during the fifth to sixth decade of life, which would approximately get diagnosed at the median age by 66 years. The incidence may vary by age; in patients above 40 years of age, approximately 18.69/100,000 is found, and this accounts for up to 43.6%, for 15–39 years, it is 15.6%, and for ages between 0–14 years, it is 1.7% of all CNS tumors (21). Approximately 70% of meningiomas occur in the thoracic vertebra, 25% in the cervical vertebra, and 5% in the thoracolumbar (12, 13, 24). Most meningiomas are intradural, and a few, accounting for up to 10%, can grow outward, resulting in tumor tissue both inside and outside the dura mater. Purely extradural spinal meningiomas are extremely rare, with an incidence rate of only 3.5% reported in the literature (4, 25).

#### Pathology and Pathogenesis

The pathogenesis of purely extradural spinal meningioma remains unclear (5). Microscopically, the typical patterns observed are meningothelial, fibroblastic, transitional, and psammomatous and most of the cases describe the existence of features resembling meningothelial or psammomatous meningioma (7, 9). An immunohistochemical demonstration has strongly diffused positive staining for epithelial membrane antigen and vimentin (24) and a low KI-67 index (4).

Usually, there are no arachnoid cap cells located extradural. Still, according to some theories, the etiology of these tumors suggests that the origin may be from (1) aberrant arachnoid islets that are positioned in the extradural space, (2) islands of arachnoidal tissue that migrated into the extradural space, (3) arachnoid villi that got separated from the main arachnoid layer and invaded into the dura, and (4) Vestigial remnants of the superficial layer of the embryonal arachnoid mater and villi from a nerve root where the dura mater is not as thick (26, 27). Pecker et al. suggested that meningiomas lying adjacent to the nerve roots could be due to the thin periradicular dura, which contained vestigial remnants of the superficial layer of the embryonal arachnoid mater and villi, and the direct fusion of spinal pia mater with the dura (28). Hassin et al. showed that the arachnoid villi present in the spinal canal occur mainly at the nerve root outlet and are therefore the favored “seats of origin” for meningiomas. Still, there is little intradural space for tumor expansion. Thus, infiltration of the dura and epidural tissues occurs, explaining the faster spread of the disease observed in earlier clinical presentations with a more aggressive nature of these lesions in these patients (29). As a thumb rule, we recommend extensive excision of the epidural adipose tissue and nerve root sheath, as well as the adjacent ventral posterior longitudinal ligament, to prevent a recurrence.

#### Clinical Signs and Symptoms

No specific differences exist in clinical symptoms between epidural meningioma and intradural meningioma (9). Initially, back or radiating root pain occurs, followed by sensorimotor changes and sphincter dysfunction. Usually, the tumor is located in the posterior or posterolateral spinal cord; it compresses the posterior corticospinal tract of the spinal cord, resulting in extrapyramidal injury and proprioceptive disorders (12), which were similar to our study results.

#### Radiographic Sign

MRI is the best non-invasive neuroimaging technique (7) that typically manifests T1W isosignal and T2W isosignal/hypersignal. Contrast enhancement shows a uniform enhancement along with the epidural meningioma located dorsally or ventrally in the dural sac. If the disease spreads rapidly, the tumor grows externally and appears as anencircle to the dural membrane or a semicircle or complete ring on the axial plane. On enhanced MRI, the so-called “dural tail sign” can be seen in epidural meningioma. However, the “dural tail sign” can also be found in metastases and lymphomas. Therefore, due to the lack of highly specific signs, it is difficult to make a definite preoperative diagnosis of epidural meningioma based on MRI alone (13). Further diagnosis through CT, the calcification in the tumor can support the diagnosis of meningioma (30). In contrast, enhanced CT can detect the blood flow in the infiltrating tissue around the tumor, mediating a safe path for biopsy by a puncture. With the development of advanced medical examination technology and diagnostic techniques in oncology, clinicians will resolve more complicated cases in the future (5).

#### Surgical Treatment

Intraspinal masses represent the potential clinical symptoms that need surgical treatment to remove all the tumors as soon as possible to achieve the purpose of spinal canal decompression. Since epidural meningiomas do not have typical clinical and radiographic features, preoperative pathological biopsy or intraoperative cryopreservation pathology is essentially needed (8). Once meningioma is identified, total resection can significantly reduce the recurrence rate (3). For most epidural meningiomas, laminectomy or hemilaminectomy can be performed. If the tumor grows laterally or ventrally, the surgical field has to be extended outwards to the facet to provide sufficient exposure to minimize the spinal displacement. A posterolateral thoracotomy may also be performed to remove the tumor anteriorly (4). If the bone structure is damaged too much, internal fixation should be performed to stabilize the alignment. In our case study, the tumor grew beyond the intervertebral foramen, and to remove the tumor at the lateral recess and foramen, the T2/T3 left facet joint was removed. Considering that the left hemilaminectomy preserves the structural integrity of the right side of the spine and the upper thoracic segment would remain stable due to the structure of the thoracic and costotransverse process joints, however, we did not use internal fixation. At the same time, if postoperative radiotherapy is needed, the effect of endoplants can be reduced.

Surgery is an optimal and effective treatment that completely removes the lesion and avoids recurrence. The main clinical factor of recurrence is due to the extent of resection (6, 18, 31), and in most cases (97%), a tumor can be totally resected (7). If complete resection is performed, there may not be any difference observed in the patient’s prognosis, while partial resection can lead to a higher probability of recurrence (24, 32). Some tumors are confined with a clear boundary consisting of the dura mater, which is helpful for their exposure and dissection. Some tumors are attached to the dura and appear as carpet-like growth, with poor tumor-dura interface, and must be incised and bluntly peeled off. Extensive paraspinal infiltration, complete envelopment of the sheath, close adhesion to the dural, and intratumoral calcification make the tumor not suitable to be completely removed (33). To avoid severe surgical complications, it is acceptable to use subtotal resection to reduce the mass effect and improve myelopathy symptoms (13). There are only a few long-term studies available on meningiomas, including the rate of late recurrence. Levy et al. reported a late relapse rate that accounted for up to 4% (34), and Solero et al. reported 1.3% (35). About 50% of patients relapse by 1 or 2 years after the surgery, and the metastasis rate is about 5% (36). Mirimanoff et al. reported that the recurrence-free rates at 5, 10, and 15 years after total resection were 93%, 80%, and 68%, respectively, while the progression-free rates during the same period after subtotal resection were only 63%, 45%, and 9% (37). Therefore, coagulation is considered an effective method to prevent recurrence (13). As the anterior dural appendages cannot be resected entirely, they must be coagulated (7). The dura mater attached to the tumor is burned or coagulated, followed by removing and reconstructing through artificial dural, which can prevent the recurrence (13). Some authors suggest that intradural exploration is a possible tumor invasion (38). However, in some cases, lymphomas, sarcomas, or metastases, a dural incision carries a risk of contamination in the cerebrospinal fluid (24). Some authors have also proposed that after the epidural mass is removed, intraoperative ultrasound can be used to detect intradural tumors or to determine whether the tumor has invaded the dural (30, 39). Microsurgery technology can find the boundary of more precise tumors and separate them from the normal tissue to be safer. The en bloc resection rate is higher, thereby reducing the risk of recurrence of the tumor. In our case study, the tumor infiltrated the epidural adipose tissue outwards, which could not be adequately separated from the nerve roots under the microscope. We decided to excise the T2 nerve root, burn and remove the nerve root sleeve, epidural adipose tissue, and the posterior longitudinal ligament to achieve the complete resection of the tumor and planned to inactivate the surrounding soft tissue. Postoperatively, gradually, the numbness in the left T2 cutaneous segment was mild but tolerable.

Although epidural meningioma is a benign tumor, the recurrence rate is higher than ordinary meningioma (40). The WHO divides meningiomas into three grades: benign (grade I), atypical (grade II), and malignant (grade III). The recurrence rate of benign meningiomas is about 7%–25%, atypical meningiomas are 29%–52%, and malignant meningiomas are 50%–94% (2, 9). For grade I epidural meningioma, the growth is slow, the risk of recurrence is low, and postoperative adjuvant radiotherapy may not be required. Adjuvant radiotherapy should be considered after resection of WHO grade II and III meningiomas (2). Some authors suggest that radiotherapy as an adjunct therapy after subtotal resection helps control unresected or recurrent meningiomas (37). However, some authors believe that the first postoperative adjuvant radiotherapy may be unnecessary because the tumor grows slowly, the risk of recurrence is low, and there is even the risk of functional spinal cord injury due to the radiation, mainly if the lesion is located in the front. The radiation is difficult to reach without exposing the spinal cord (13). Advances in neuroradiology and neurosurgery have improved the surgical outcomes for spinal tumors. The accepted recommendations of adjuvant radiotherapy are as follows: (1) after total or subtotal resection of early recurrence; (2) when total resection is not possible due to the tumor’s location or the patient's medical condition, (3) when the medical risks of surgical treatment are high, reoperation should be performed in case of early recurrence, followed by radiotherapy (13). Some authors have suggested that if the regenerative tumor cannot be removed and the nerve function gradually deteriorates, then radiotherapy, especially stereotactic radiosurgery, such as CyberKnife, can minimize the risk of radiological complications and should be considered a useful therapeutic option (41). Electrophysiological monitoring helps dissect the tumor from the nerve root components (24). The development of microsurgery technology increases the scope of tumor resection and reduces the mortality and recurrence rate of meningiomas. The location of the tumor is no longer a factor affecting the prognosis (6, 13, 42).

## Conclusion

Purely extradural spinal meningioma with nerve root attachment is rare and has no characteristic clinical symptoms or image findings. To completely remove the lesion and avoid recurrence, the affected nerve root, epidural fat tissue, and nerve root sheath should be extensively resected and burned, followed by coagulating the adjacent ventral posterior longitudinal ligament. Microsurgical techniques can improve the tumor resection rate, increase the safety of normal tissue, and reduce the risk of recurrence. A preoperative or intraoperative pathological biopsy is significant for diagnosing and treating this disease.

## Data Availability

The original contributions presented in the study are included in the article/Supplementary Material, further inquiries can be directed to the corresponding author/s.
